# Spectrum of EGFR gene mutations and ALK rearrangements in lung cancer patients in Turkey

**DOI:** 10.1186/s40064-016-2150-4

**Published:** 2016-04-19

**Authors:** Sebnem Ozemri Sag, Ozlem Gorukmez, Mehmet Ture, Orhan Gorukmez, Adem Deligonul, Serdar Sahinturk, Ali Topak, Tuna Gulten, Ender Kurt, Tahsin Yakut

**Affiliations:** Department of Medical Genetics, Faculty of Medicine, Uludag University, 16059 Görükle, Bursa Turkey; Department of Medical Genetics, Sevket Yılmaz Education and Research Hospital, 16310 Yıldırım, Bursa Turkey; Division of Medical Oncology, Department of Internal Medicine, Faculty of Medicine, Uludag University, 16059 Görükle, Bursa Turkey

**Keywords:** Lung cancer, Non-small cell lung cancer, EGFR mutations, ALK rearrangement

## Abstract

The EGFR gene and ALK rearrangements are two genetic drivers of non-small cell lung cancer (NSCLC). The frequency of EGFR mutations and ALK rearrangement varies according to not only ethnicity but also gender, smoking status and the histological type of NSCLC. In the present study, we demonstrated the distribution of EGFR mutations in 132 NSCLC patients by using a pyrosequencing technique and the distribution of ALK rearrangements in 51 NSCLC patients by using fluorescent in situ hybridization technique in Turkey. Additionally, we compared the clinicopathological data of NSCLC patients with the mutation status of EGFR in their cancerous tissues. Both EGFR mutations and ALK rearrangements were identified in 19 (14.39 %) and 1 (1.96 %) patients, respectively. We found EGFR mutations in codon 861, 719 and 858 with the ratios of 10.52 % (2/19), 10.52 % (2/19) and 31.58 % (6/19), respectively, and deletion of exon 19 in 47.37 % (9/19) of the patients. We found the frequency of EGFR mutations to be significantly higher in female patients and nonsmokers (p = 0.043, p = 0.027, respectively). Consequently, we found EGFR mutations to be more frequent in female patients and nonsmokers. Future studies on larger patient groups would provide more accurate data to exhibit the relationship between EGFR mutations and ALK rearrangements and the clinicopathological status.

## Background

Lung cancer is the most common cause of cancer-related deaths in males and females worldwide (Jemal et al. 2010). Non-small cell lung cancer (NSCLC) accounts for 80–85 % of all lung cancers. Most of the patients have advanced stages or metastatic disease (D’Addario et al. 2010; Crinò et al. 2010). A recent improvement in comprehending the molecular basis of the disease, especially NSCLC, has led to improvements in treatment (Mok et al. 2009). In recent years, because of the development of biomarker-driven personalized therapy, the treatment approach for many cancers including the NSCLC has changed (Vagulienė et al. 2012). Various phase 3 trials have demonstrated the clinical efficacy of EGFR tyrosine kinase inhibitors (gefitinib and erlotinib) as first-line therapies compared with chemotherapy in advanced stage NSCLC patients with harboring activating EGFR mutations. Some clinical practice guidelines suggest testing EGFR mutation status before initiating the first-line treatment to patients with advanced stage NSCLC (Shi et al. 2014). The EGFR gene is localized on the short arm of chromosome 7 and encodes a 170-kDa type I transmembrane growth factor receptor. The *EGFR* receptor belongs to the HER/erbB family of tyrosine kinase. This receptor has an extracellular cysteine-rich ligand-binding domain and an intracellular domain which has intrinsic tyrosine kinase activity. Intracellular signaling is mediated mainly by the RAS–RAF–MEK–MAPK pathway, PI3K–PTEN–AKT pathway and the signal transducer and activator of transcription (STAT) pathways (De Luca et al. 2008). These EGFR signaling pathways are important for tumor cell growth, local invasion, angiogenesis, protein translation, autophagy and cell metabolism (Jimeno and Hidalgo 2006). Echinoderm microtubule-associated protein-like 4 and the anaplastic lymphoma kinase (EML4–ALK) fusion genes were identified in a subset of NSCLCs in 2007 (Soda et al. 2007). This fusion occurs due to chromosomal inversion or the translocation of chromosome 2 p and results in the formation of EML4–ALK fusion gene.

The EML4–ALK translocation results in constitutive ALK kinase activity and represents the oncogenic addiction pathway in NSCLC (Soda et al. 2007, 2008). The ALK rearrangements that are found in up to 5 % of patients with NSCLC are related to younger age, no history of smoking and distinct clinicopathological features such as adenocarcinoma histology (Shaw et al. 2009; Solomon et al. 2009; Tiseo et al. 2011; Wong et al. 2009). Crizotinib, a targeted ALK tyrosine kinase inhibitor (TKI), is a promising agent in treating ALK-positive NSCLC patients and provides survival benefit (Kwak et al. 2010). It was approved by the US Food and Drug Administration (FDA) for treating advanced stage ALK-positive lung cancer.

The aim of the study was to examine the prevalence of EGFR-activating mutations and ALK rearrangements among patients with NSCLC and to evaluate the associations between EGFR mutations and clinicopathological characteristics.

## Methods

One hundred and thirty two patients applying to Uludağ University Faculty of Medicine, Medical Genetics Department for EGFR mutation analysis and 51 patients applying for ALK translocation analysis with histopathologically proven NSCLC were enrolled in this retrospective study. The demographic (age, gender) and clinicopathological characteristics of the patients (smoking status, histological type and metastatic status) were compared with the results of molecular analysis. Approval for the study was granted by the local ethics committee.

### EGFR mutation analysis using pyrosequencing

Formalin-fixed paraffin embedded tissue samples were obtained by tumor biopsy. The slides were examined by pathologists to confirm that they contain more than 50 % of tumoral tissue, and they are appropriate for DNA extraction. The QIAamp^®^ DNA FFPE tissue kits were used to extract the human DNA from formalin-fixed paraffin-embedded tissue specimens (QIAGEN^®^ kit, Germany). Single-stranded DNA was prepared, and the corresponding sequencing primers were annealed to the DNA. Therascreen EGFR Pyro Kit can detect and measure the mutations in human EGFR gene 719, 768, 790, and 858–861 codons quantitatively and also detect the deletions and complex mutations in exon 19. The kit includes 4 PCR assays: one for detecting the mutations in codon 719 (exon 18), one for 768 and 790 (exon 20), one for codons 858–861 (exon 21) and one for the deletions in exon 19. Four regions were amplified separately and sequenced through the defined region. After using primers targeting 18, 19, 20 and 21, amplicons were immobilized on Streptavidin Sepharose High Performance beads. The samples were analyzed in a PyroMark Q24 system using Assay setup files and a run setup file. The unmethylated control DNA was included in the study as a positive control for the PCR and sequence analyses. Additionally, negative controls (without template DNA) were included per each PCR set up for at least one assay.

### Fluorescent in situ hybridization (FISH) for EML4–ALK fusion

Formalin-fixed, paraffin-embedded tissue sections were localized on slides. The DNA was denatured to a single-stranded form and was hybridized with DNA probes. Following the hybridization, the undyed probes were removed by a washing series, and the nuclei were counter-stained with DAPI (4,6 diamidino-2-phenylindole) (a DNA-specific stain that fluoresces blue). The hybridization of ALK probe (Vysis LSI ALK Dual Color, break-apart rearrangement probe; Abbott Molecular, Abbott Park, IL) was examined using a fluorescence microscope equipped with appropriate excitation and emission filters, allowing the visualization of the orange and green fluorescent signals. If 2p23 ALK region is hybridized with Vysis ALK Break Apart FISH Probes, it appears as two adjacent or fused (overlapping) orange/green (yellow) signals in a natural position. However, if a chromosome rearrangement at the 2p23 ALK breakpoint region has occurred, one orange and one green signal separated by at least two signal diameters will be visualized. Alternatively, a single orange signal (deletion of green signal) in addition to a fused or broken apart signal may be seen. If more than 100 tumor cells are counted, if >15 % of tumor cells showed a split red and green and/or single red pattern, then the occurrence of an ALK gene rearrangement is considered; otherwise, the specimen is classified as *ALK* FISH negative. The ALK positivity pattern (split, single red or both) was recorded.

### Statistical analysis

The age variable was presented with median value (minimum–maximum), and the other categorical variables of the study were presented in numbers and percentages. The Pearson’s Chi square and Fisher’s exact Chi square tests were used for the comparisons of categorical variables between mutation groups. Analyses were performed with SPSS 22.0 (Chicago, IL) program, and p < 0.05 was considered statistically significant.

## Results

The median age of 132 patients with NSCLC diagnosis was 60 (31–83) years. The demographic and clinicopathological characteristics of the patients are given in Table 1. EGFR mutation and ALK rearrangements were detected in 14.39 % (19/132) and 1.96 % (1/51) of the patients, respectively. We found EGFR mutations in codon 861, 719, and 858 in the following ratios: 10.52 % (2/19), 10.52 % (2/19) and 31.58 % (6/19) and detected exon 19 deletion in 47.37 % of the patients (9/19). Among all mutations, we found exon 19 deletions delE746-A750 and delL747-p753, InsS with ratios of 42.11 % (8/19) and 5.26 % (1/19), respectively. In one patient with codon 719 mutation, we also detected a mutation at codon 790. The comparison of clinicopathological characteristics of EGFR mutation carriers and wild type patients is presented in Table 1. The patients were classified as adenocarcinoma and the others (squamous, giant cell, adenosquamous) in terms of histological type.

**Table 1:** The demographic and clinicopathological characteristics of the patients and the comparison of clinicopathological characteristics of EGFR mutation carriers and wild type patients

Characteristics	All patients (n = 132)	Patients with EGFR mutation (n = 19)	Patients with EGFR wild type (n = 113)	p value
*Age*				0.556
<65	91 (68.9 %)	12 (13.2 %)	79 (86.8 %)	
≥65	41 (31.1 %)	7 (17.1 %)	34 (82.9 %)	
*Gender*				0.043
Male	98 (74.2 %)	10 (10.2 %)	88 (89.8 %)	
Female	34 (25.8 %)	9 (26.5 %)	25 (73.5 %)	
*Smoking status*				0.027*
Never	60 (45.5 %)	13 (21.7 %)	47 (78.3 %)	
Ever	56 (42.4 %)	4 (7.1 %)	52 (92.9 %)	
Unknown	16 (12.1 %)	2 (12.5 %)	14 (87.5 %)	
*Histology*				0.546
Adenocarcinoma	127 (96.2 %)	18 (14.2 %)	109 (85.8 %)	
Others	5 (3.8 %)	1 (20 %)	4 (80 %)	
*Metastasis status*				0.780
Present	98 (74.2 %)	15 (15.3 %)	83 (84.7 %)	
Absent	34 (25.8 %)	4 (11.8 %)	30 (88.2 %)	

*EGFR* epidermal growth factor receptor

* p value were derived from a comparison between never and ever smoking status

EGFR mutation rates were higher in females than males (p = 0.043) and higher in non-smokers than smokers (p = 0.027). We did not find additional significant associations between EGFR mutation and patients’ clinicopathological characteristics (Table 1). Clinicopathological characteristics of a 54 year old female patient who had ALK rearrangements and EGFR wild type was smoker, adenocarcinoma histology and presence of metastasis.

## Discussion

The molecular basis of lung cancer is complex and heterogeneous. The molecular changes in multiple levels (genetic, epigenetic, protein expression) and the improvements in the comprehension of the functional expressiveness of these changes have potential effects for the diagnosis, prognostication and treatment of lung cancer. EGFR mutations are related to the pathogenesis of many types of cancers including NSCLC (Cooper et al. 2013). Activating mutations of the EGFR gene were reported in 10–15 % of unselected Western patients (Yip et al. 2013; Shigematsu et al. 2005; Eberhard et al. 2008; Russell et al. 2013) and 30–40 % of Asian populations (Kosaka et al. 2004; Tokumo et al. 2005; Yoshida et al. 2007). EGFR mutations in NSCLC are seen in the first 4 exons of intracellular tyrosine kinase domain. They are most commonly frame deletions of exon 19 (~45 %) and have over 20 variants, and the most common of which is delE746-A750. The next most common mutations are missense mutations, particularly L858R in exon 21, a single nucleotide point mutation leading to a single amino acid change from leucine to arginine at codon 858 (~40 %). However, Yip et al. reported that 14 % of the EGFR mutations of patients with early stage lung cancers comprised activating mutations of exon 18 and that L858R mutations accounted for only 29 % of EGFR mutations in an Australian population (Yip et al. 2013). Additionally, there are less common types of mutations including the frame duplications and insertions in exon 20 (~5–10 %), most of which are related to EGFR-TKI resistance (Tam et al. 2006; Yamamoto et al. 2009). Doğan et al. reported that all of the EGFR mutations they detected in 7.4 % of 42 patients with advanced stage NSCLC were exon 19 deletions (Dogan et al. 2014). Shigematsu and Gazdar (2005), one of two major series (Shigematsu and Gazdar 2005; Murray et al. 2008) on epidemiology of EGFR mutations stated that all the reported mutations were somatic mutations at exon 18–21 of EGFR gene encoding a part of intracellular TK domain. Three different mutation types related to EGFR-TKIs sensitivity were defined: (1) in-frame deletions at exon 19 which are the most common mutations accounting for 46 % of EGFR mutations, (2) missense mutations at exon 18, 20 or 21 (primarily the single-nucleotide substitutions, L858R at exon 21) that are the second most common mutations accounting for 41 % of EGFR mutations and (3) in-frame duplications/insertions at exon 20 that constitute 5 % of all EGFR mutations. It was found that EGFR mutations are associated with EGFR-TKIs sensitivity as well as East Asian ethnicity, female gender, no history of smoking and adenocarcinoma histology.

Murray et al. (2008) screened the data of 12,244 patients and noted 3381 EGFR mutations. Exon 19 mutations were the most common (50 %) followed by exon 21 (40 %), 20 (6 %), and 18 mutations (4 %). Whereas L858R and delE746-A750 constituted 32.8 and 24.3 %, respectively, 50 % of the mutations were deletions of exon 19 or deletion-insertions. There was a significant relationship between TKI response and presence of mutation, and although exon 19 mutations have the best response rates (70 %), exons 21, 18 and 20 have a response rate of approximately 20 % or slightly more (Shigematsu and Gazdar 2005; Murray et al. 2008). Ünal et al. found EGFR mutations in 42.6 % of 48 patients with NSCLC in their study in Western Turkey in which 9 of the mutations were at exon 20; 7 were at exon 19 and 2 were at exon 21 (Unal et al. 2013). In the present study, we detected EGFR mutations in 14.39 % (19/132) of NSCLC patients, and the mutations were in codons 861, 719 and 858 with the following rates: 10.52 % (2/19), 10.52 % (2/19) and 31.58 % (6/19), respectively, and deletion of exon 19 was seen in a ratio of 47.37 % (9/19). delE746-A750 accounted for 42.11 % of all mutations, and it was the most common mutation. Our results were consistent with the literature.

The data from the literature indicate that the mutations are more frequent in females compared with males (42 vs. 14 %), in non-smokers compared with smokers (51 vs. 10 %) and in patients with tumor histology of adenocarcinoma compared with other types (40 vs. 3 %) (Jimeno and Hidalgo 2006; Paez et al. 2004; Lynch et al. 2004; Pao et al. 2004; Shigematsu and Gazdar 2006; Mitsudomi et al. 2006; Jänne et al. 2004; Sakurada et al. 2006). Additionally, Vaguliene et al. found that EGFR mutations were significantly more frequent in females and nonsmokers in a study of 103 patients with NSCLC (Vagulienė et al. 2012). Ünal et al. found that EGFR mutations were significantly more frequent in non-smoking NSCLC patients in Western Turkey (Unal et al. 2013). In contrast to majority of published data, Skov et al. (2015) and Hsiao et al. (2014) found no difference in mutation rate among men and women. In our study, we found that EGFR mutations were more common in females and non-smokers.

In former studies, ALK rearrangement frequency was reported to be 3–13 % using FISH technique in NSCLC patients (Shaw et al. 2009; Inamura et al. 2008; Lin et al. 2009). In those studies, it was stated that ALK rearrangements were more common in nonsmoking patients, younger patients and adenocarcinoma histology and that it rarely coincided with the presence of an EGFR mutation (Shaw et al. 2009; Wong et al. 2009; Takahashi et al. 2010). Wang et al. detected ALK rearrangements in 9.6 % (32/332) of 332 patients with primary lung adenocarcinoma (Wang et al. 2014). In the present study, we found ALK rearrangement in 1.96 % (1/51) of our patients; however, we could not establish an association with clinicopathological parameters due to a limited number of patients with ALK rearrangements.

A few weaknesses of the study should be mentioned. First, this is a single center study. Second, our study was performed on a relatively small population. Finally, although the cohort is complete and genetically homogenous, the mutation rate is dependent on the prevalence of smoking.

## Conclusions

We detected EGFR mutation and ALK rearrangements at proportions of 14.39 and 1.96 %, respectively. We found that EGFR mutations were significantly more common in females and nonsmokers. The distinctness in prevalence reported in various studies may be related to differences in patient populations and may also stem from the diversity in sensitivity of the techniques to analyze mutations. Further studies in larger patient groups would provide more accurate information to exhibit the association between clinicopathological characteristics and EGFR mutations and ALK rearrangements.
